# Understanding Delays in Breast Cancer Diagnosis in Bangladesh: A Facility‐Based Cross‐Sectional Study

**DOI:** 10.1002/hsr2.72718

**Published:** 2026-06-28

**Authors:** Mohammad Sorowar Hossain, Mohammad Nayeem Hasan, Sumaya Khan Trisha, Md. Waheed Akhter

**Affiliations:** ^1^ Department of Emerging and Neglected Diseases Biomedical Research Foundation Dhaka Bangladesh; ^2^ School of Environment and Life Sciences Independent University, Bangladesh Dhaka Bangladesh; ^3^ National Institute of Cancer Research & Hospital Dhaka Bangladesh

**Keywords:** Bangladesh, breast cancer, cancer staging, clinical characteristics, delays in diagnosis

## Abstract

**Background and Aims:**

This study investigates factors contributing to delays in breast cancer diagnosis in Bangladesh and their impact on cancer staging. Early detection is crucial for effective treatment, yet many women in low‐ and middle‐income countries (LMICs) are diagnosed at advanced stages, resulting in poorer outcomes.

**Methods:**

A cross‐sectional study was conducted at two major cancer care facilities in Dhaka. Women aged 18 and older with suspected or confirmed breast cancer were included. Data were collected using a structured questionnaire on sociodemographic and clinical variables. Total delay, defined as the time from symptom recognition to treatment initiation, was categorized into patient delay (symptom recognition to first medical consultation) and provider delay (first consultation to treatment start). Multivariable logistic regression analyses identified factors associated with these delays.

**Results:**

Among 355 participants, 55.7% experienced total delays of over 4 months, with the highest delays in stage III cases (51.5%) due to patient delays. Key factors contributing to patient delay included low education (AOR: 1.43, 95% CI: 1.17–3.05), low monthly income (AOR: 3.41, 95% CI: 1.65–7.30), and absence of breast pain (AOR: 0.50, 95% CI: 0.26–0.96). Provider delays were significantly associated with rural residence (AOR: 3.07, 95% CI: 1.49–6.98) and the presence of nipple discharge (AOR: 2.92, 95% CI: 1.04–8.06). Total delays were most prevalent among patients from the Rangpur division (AOR: 5.87, 95% CI: 2.11–6.59), those residing in rural areas (AOR: 2.74, 95% CI: 1.40–5.52), and individuals with low education (AOR: 2.33, 95% CI: 1.09–5.11). Clinical factors such as breast pain (AOR: 2.34, 95% CI: 1.22–4.60) were also significantly associated with delay, along with discomfort discussing symptoms with a spouse (AOR: 2.16, 95% CI: 1.03–4.68). Additionally, nearly 80% of patients delayed seeking medical attention due to the belief that symptoms would resolve spontaneously, while 75% cited negligence and 65.5% reported financial barriers.

**Conclusions:**

Significant delays in breast cancer diagnosis in Bangladesh are driven by socio‐economic factors and inadequate healthcare access. Increasing public awareness, especially in rural areas, and improving healthcare accessibility are essential to facilitate early detection. Expanding screening programs and training healthcare providers in early cancer detection are critical to improving patient outcomes.

**Abbreviations**
AOR: adjusted odds ratio
CI: confidence interval
COR: crude odds ratio
LMICs: low‐ and middle‐income countries
NGOs: non‐governmental organizations
NICRH: National Institute of Cancer Research and Hospital
TNM: tumor, node, and metastasis
VIF: variance inflation factor

## 1 Background

Breast cancer is a significant public health concern worldwide, with its impact expected to rise. In 2020, approximately 685,000 women died from breast cancer, accounting for around 16% of all cancer‐related deaths among females—equivalent to one in every six cancer fatalities [1]. While the incidence of breast cancer is lower in developing countries compared to their developed counterparts, the mortality rates are disproportionately higher in these regions [2].

Although the incidence is rising due to epidemiological transitions in low‐ and middle‐income countries (LMICs), breast cancer mortality is projected to increase by 53.6% between 2020 and 2040 [3]. This alarming trend poses significant challenges for policymakers aiming to reduce breast cancer mortality. A critical factor contributing to the higher mortality rates is delayed diagnosis, often exacerbated by limited access to healthcare [4]. Delayed diagnosis can be more detrimental to patient survival than the disease itself, as early detection and treatment are linked to improved prognoses [5]. Additionally, delays may lead to advanced disease stages and larger tumors.

In LMICs, two specific types of delays are particularly concerning. Patient delay refers to the interval between the onset of symptoms and the decision to seek medical attention. Research indicates that this delay often results from factors such as lack of awareness, educational deficits, cultural barriers, and fear of diagnosis [6, 7, 8, 9]. Conversely, treatment/provider delay is the period between diagnosis and the initiation of treatment, influenced by healthcare system limitations such as inadequate primary care resources and ineffective referral processes [10]. Both types of delays have been shown to negatively impact patient outcomes; a meta‐analysis by Hanna et al. revealed that a 4‐week treatment delay post‐diagnosis significantly increases mortality risk in breast cancer patients [11]. Therefore, addressing these delays through enhanced awareness, improved healthcare access, and strengthened healthcare systems is crucial for improving survival rates.

In Bangladesh, breast cancer represents a significant health disparity. According to the 2020 GLOBOCAN report, breast cancer is the leading cause of cancer deaths among women, responsible for 6.2% of all cancer‐related fatalities and accounting for 19% of all female cancer cases [12]. These statistics underscore the substantial burden of breast cancer on women's health and the urgent need for targeted healthcare interventions. Recent studies in Bangladesh have highlighted persistent gaps in awareness, screening practices, and healthcare access, particularly among rural and low‐income populations [13, 14]. Cancer prevention in Bangladesh depends on strong political support and stable leadership to ensure funding and effective policies, while economic limitations and weak healthcare infrastructure restrict service delivery, especially in rural areas. Additionally, demographic factors such as low literacy, cultural beliefs, and social norms influence awareness and participation, meaning interventions must be locally adapted to improve outcomes and reduce inequalities [13]. A study on cancer risk among Bangladeshi women found that employment status is associated with breast cancer subtypes, suggesting that this relationship should be interpreted in the context of Bangladesh's socioeconomic conditions, including occupational stress, environmental exposures, and disparities in access to reproductive healthcare [14].

Women play a vital role in Bangladesh's economy and social development, particularly in sectors such as clothing and microfinance. Their health is essential for fostering healthy families and communities. However, issues related to women's health, including breast cancer, often receive insufficient attention. Alarmingly, about 90% of breast cancer patients in Bangladesh are diagnosed at advanced stages (III and IV) [15]. Delayed diagnosis significantly worsens outcomes and reduces survival rates, while early detection improves prognosis and lowers treatment costs [16, 17]. Therefore, reducing diagnostic delays is critical for improving health outcomes.

A number of factors, including age, education, occupation, living in a rural area, consulting a traditional healer, armpit lumps, and other medical conditions, can contribute to the delayed presentation of breast cancer [18]. Due to a lack of awareness, a lack of understanding of available treatments, and uncertainty about where to seek care, many patients receive their diagnoses later than they should [19]. Almost one‐fourth of those surveyed were unsure of who to start with. Many first resort to alternative medicine because of its accessibility, affordability, and low literacy rates [20, 21]. Significant obstacles prevent rural women, particularly those from lower socioeconomic backgrounds, from receiving timely diagnosis and care, which causes additional delays [22, 23, 24].

Despite the fact that breast cancer is becoming a more significant public health issue in Bangladesh, little is known about early detection. Although we published the nation's first thorough review of breast cancer in 2014 [15], there hasn't been much progress in addressing diagnostic delays. Recent research has examined perceived barriers to screening [25] or general awareness and knowledge among particular groups, such as female university students [26]. These studies, however, do not fully investigate the underlying reasons for delayed diagnosis or take into account factors at the patient and provider levels. The lack of a comprehensive understanding of diagnostic delays is a major gap, since early detection is essential for bettering results. There is currently no systematic analysis of the ways in which cultural beliefs, healthcare system inefficiencies, and socioeconomic circumstances interact to postpone diagnosis in Bangladesh. However, limited research has simultaneously examined patient‐level, sociocultural, and health system determinants of delay in a single analytical framework. This study aims to examine the socioeconomic and health system‐related factors contributing to diagnostic delays in breast cancer from both patient and provider perspectives. The results are intended to guide focused interventions and policy plans to shorten diagnostic wait times and enhance the prognosis of breast cancer in Bangladesh.

## 2 Methods

We adhered to the STROBE guidelines to ensure high‐quality reporting in our observational cross‐sectional study (Supporting Information S1: Table S1).

### 2.1 Study Site

This cross‐sectional study was conducted at the National Institute of Cancer Research and Hospital (NICRH), the only public facility in Bangladesh dedicated exclusively to cancer treatment. Public hospitals, like NICRH, primarily serve individuals from economically disadvantaged and lower‐middle‐class backgrounds, as the costs of treatment in private facilities are often unaffordable for most Bangladeshis [15]. Currently, Bangladesh lacks organized breast cancer screening programs, leading to almost all cases being diagnosed through clinical evaluation rather than early detection. Additionally, unlike in developed countries, there is no systematic referral system in place, and medical record‐keeping is inadequate.

### 2.2 Patients

The study focused on women over 18 years old who presented with suspected breast cancer or had been diagnosed with the disease. Only those patients were enrolled whose initial cancer stage was documented in their medical records or, in cases where staging was unavailable, if the initial diagnosis occurred no more than 6 months prior to staging at our study center.

### 2.3 Questionnaire

We adapted a structured questionnaire from previous studies [5, 27]. The questionnaire comprised sections on sociodemographic variables, including age, education level, marital status, residence, and access to media and electronic devices. It also collected clinical history regarding breast cancer symptoms, capturing the type of initial symptoms (e.g., lump, breast pain, nipple discharge), the date of first symptom recognition, and participants' perceptions of their symptoms' severity.

Furthermore, the questionnaire explored barriers to seeking care, encompassing emotional factors (e.g., fear, embarrassment), practical constraints (e.g., financial limitations, time constraints), and health service‐related issues (e.g., challenges in accessing healthcare, arranging transportation, or scheduling appointments). Participants were asked about their healthcare utilization, including the type of medical facility they first visited and any alternative treatments sought prior to diagnosis. The survey also assessed family support by gathering information on initial discussions about health concerns, recommendations to seek medical attention, and the level of support received after diagnosis. Knowledge and practices related to early detection were evaluated, focusing on breast self‐examinations, prior clinical breast examinations, and awareness of mammography. Clinical variables, including tumor size and cancer stage classified by the tumor, node, and metastasis (TNM) system, were recorded. The data collected to analyze associations between these variables and delays in diagnosis, offering insights into factors contributing to late‐stage detection and their potential impact on treatment outcomes. The questionnaire was finalized for data collection after piloting it with five patients. The data collected during the pilot phase were consistent with the main study objectives and met the quality criteria for inclusion.

### 2.4 Data Collection

A convenience sampling method was employed due to the unavailability of patient registries. Eligible participants were patients presenting at the participating facilities during the study period who met the predefined inclusion and exclusion criteria. Data collection was conducted from April 2017 to September 2017. Prior to participation, verbal informed consent was obtained in accordance with the approved study protocol. Structured, face‐to‐face interviews were carried out by trained final‐year female undergraduate students who were not involved in the clinical management of the patients. Considering the conservative cultural context, all interviewers were female and worked under the close supervision of the team's oncologist at the National Institute of Cancer Research & Hospital (NICRH).

Participants included women aged 18 years and older who had either been diagnosed with breast cancer or were suspected cases referred to the participating centers. Clinical data related to symptom onset, first medical consultation, and diagnosis were extracted from patients' medical records where available. In cases where specific dates could not be recalled, participants were asked to provide an approximate month or range of months and the year. If a single month was given, the 15th day of that month was recorded; for a range of months, the midpoint between the 15th of each month was used. When only the year was recalled, the date was recorded as 30th June of that year. Cancer staging was determined by the team's oncologist based on available medical documentation. However, staging could not be determined for 355 patients due to insufficient clinical records.

### 2.5 Outcome Variables

In this study, delay is defined as the time interval experienced by women in the diagnostic and treatment processes. Patient delay refers to the time between the onset of symptoms and the first medical consultation, with a commonly accepted threshold for defining this delay being 3 months. Provider delay, or system delay, refers to the time that passes between the initial medical consultation and the final diagnosis or treatment, with a commonly accepted threshold of 1 month [5]. Total delay encompasses the entire duration from the patient's first recognition of symptoms to the start of definitive treatment, integrating both patient and provider delays. In our study, total delay is considered significant when it exceeds 4 months. To quantify our outcome variables related to delays, we categorized each patient as “1” or “Yes” if they experienced patient delay, provider delay, or total delay that surpassed the thresholds of 3 months, 1 month, and 4 months, respectively. Conversely, patients who did not meet these criteria were recorded as having no delays, designated by “0” or “No.”

Data were collected on factors associated with patient delays, such as believing the problem would resolve on its own, fear of cancer diagnosis and/or treatment, financial constraints, competing life priorities, embarrassment about breast examinations, negligence or carelessness, etc. Additionally, system‐related delays included appointment delays, misinterpreted mammography, difficulty arranging transport, lack of information, etc.

### 2.6 Possible Factors

To find out potential risk factors associated with various types of delays, we examined a range of socioeconomic factors and the medical history of the patients as independent variables. These included the patient's age, geographic location (division), residency (urban or rural), educational attainment (illiterate, primary, and secondary) of both the patient and their spouse, household monthly income, access to portable electronic devices, exposure to mass media, lump breast pain, nipple discharge, skin changes, bone pain, breast self‐examination, and family history of breast cancer. Additional behavioral and sociocultural variables included use of alternative or home‐based treatment, discomfort discussing symptoms with spouse, history of prior clinical breast examination, and type of first‐visited healthcare facility for breast examination.

### 2.7 Statistical Analyses

We conducted descriptive statistics and also differences between delays associated with other factors tested by *χ* ^2^ tests and Fisher's exact test (in case of low frequency). Univariable (unadjusted) and multivariable (adjusted) logistic regression were utilized to identify factors that are associated with patient delay, provider delay, and total delay. In the univariable analysis, variables were individually added to the logistic regression model. We used a *p* value threshold of ≤ 0.20 in univariable analyses as a screening criterion for inclusion in multivariable models to avoid excluding potentially important covariates, as commonly recommended in epidemiological modeling [28].

In this study, three models were utilized to identify associated risk factors of patient delay, provider delay, and total delay, designated as Model 1, Model 2, and Model 3, respectively. Results were reported as unadjusted/crude odds ratios (COR) and adjusted odds ratios (AOR) with their respective 95% confidence intervals. All statistical tests were two‐sided and we considered a *p* value of less than 0.05 to be statistically significant, indicating a 5% level of significance for interpreting our results. Additionally, we assessed multicollinearity in the final model using a cut‐off value of 4.00 for the variance inflation factor (VIF) analysis [29]. At this stage, all variables were incorporated into the model since the VIF values for each variable were below 4.00. All statistical analyses were conducted using R software (version 4.5.3).

## 3 Results

### 3.1 Study Population

A total of 355 women participated in our study. The largest age group was 40‐49 years, comprising 35.0% of participants. Most women resided in the Dhaka division (44.0%), with a significant proportion (72.8%) from rural areas. A notable majority of participants were married (82.8%). Illiteracy was prevalent, affecting 42.9% of patients, while primary education was the most common level of education among spouses (37.1%). Over a third of families reported a monthly income of less than 5000 BDT. Access to electronic devices was high (89.9%), but 59.4% lacked access to mass media (Table 1).

**Table 1:** Characteristics of women diagnosed with breast cancer and of their spouses, Bangladesh (*N* = 355).

Characteristics	*n*	%
Age at presentation (years)		
< 40	114	33.24
40–49	120	34.98
50–59	79	23.03
≥ 60	30	8.75
Regions of origin		
Barisal	26	7.67
Chittagong	52	15.34
Dhaka	149	43.95
Khulna	39	11.50
Mymensingh	33	9.73
Rajshahi	21	6.19
Rangpur	14	4.13
Sylhet	5	1.47
Area of residence		
Rural	251	72.75
Urban	94	27.25
Current marital status		
Single	59	17.25
Married	283	82.75
Patient education level		
Illiterate	147	42.86
Primary	115	33.53
Secondary/higher	81	23.62
Spouse education level		
Illiterate	89	27.99
Primary	118	37.11
Secondary/higher	111	34.91
Household monthly income (BDT)		
< 5000	113	34.88
5000–10,000	100	30.86
10,001–20,000	67	20.68
> 20,000	44	13.58
Portable electronic devices access		
Yes	319	89.86
No	36	10.14
Mass media access		
Yes	144	40.56
No	211	59.44

### 3.2 Clinical Characteristics and Associated Factors

As shown in Tables 2, 88.5% of patients initially presented with a lump. Only 11.1% practiced breast self‐examination, and 9.5% reported a family history of breast cancer. Patient delay (defined as a delay of more than 3 months) occurred in 41.0% of cases, while provider delay (more than 1 month) was noted in 24.3%. Total delay (more than 4 months) affected 55.7% of participants. Most patients sought medical help at stage II (48.8%) or stage III (44.1%).

**Table 2:** Medical history of the patients.

Characteristics	*n*	%
First clinical presentations^a^		
Lump	314	88.45
Breast pain	93	26.20
Nipple discharge	20	5.63
Skin changes	15	4.23
Bone pain	12	3.38
Breast self‐examination	37	11.11
Family history of breast cancer	32	9.52
Patient delay	139	41.00
Provider delay	82	24.33
Total delay	190	55.72
Stage of cancer		
Stage I	10	3.94
Stage II	124	48.82
Stage III	112	44.09
Stage IV	8	3.15

^a^ multiple answer.

**Figure 1:**
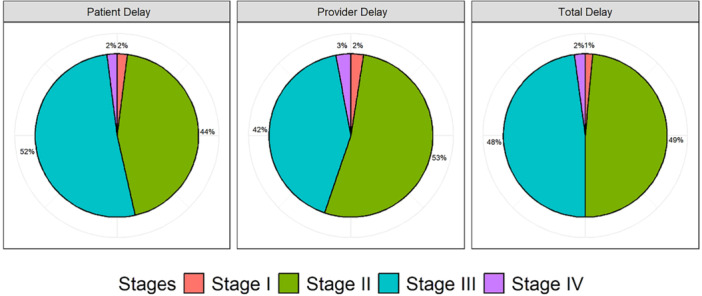
Breast cancer delays at various stages.

Statistical analysis revealed significant differences in patient delay across cancer stages, with mean delays of 0.93 months for stage I, 5.23 months for stage II, 6.33 months for stage III, and 3.21 months for stage IV. Provider and total delays did not show significant variation across stages. Notably, stage III exhibited the highest patient delay (51.5%) and total delay (48.6%), while stage I had the lowest patient delay (2.0%) and total delay (1.4%). These findings underscore the urgent need for targeted interventions to reduce delays at various cancer stages (Table 3 and Figure 1).

**Table 3:** Summary of patient delay, provider delay, and total delay with stage of cancer (in weeks).

	Patient delay					
Stage of cancer	Mean	Standard deviation	Median	Minimum	Maximum	*p* value^a^
Stage I	0.93	1.89	0.07	0.00	6.00	0.022
Stage II	5.23	8.60	2.00	0.00	48.67	
Stage III	6.33	10.22	3.00	0.00	60.83	
Stage IV	3.21	3.80	2.50	0.00	12.17	
Total	5.69	9.23	2.00	0.00	60.83	
	Provider delay					
Stage I	1.80	2.55	0.53	0.00	6.57	0.973
Stage II	1.75	2.50	0.53	0.00	7.20	
Stage III	1.40	2.10	0.48	0.00	7.13	
Stage IV	1.22	2.04	0.40	0.00	6.00	
Total	1.49	2.25	0.47	0.00	7.20	
	Total delay					
Stage I	2.73	3.93	0.93	0.03	12.57	0.120
Stage II	6.89	8.52	5.70	0.00	48.67	
Stage III	7.73	10.14	5.92	0.00	61.33	
Stage IV	4.43	4.01	3.67	0.13	12.17	
Total	7.13	9.22	5.40	0.00	66.83	

^a^ Kruskal–Wallis test.

**Figure 2:**
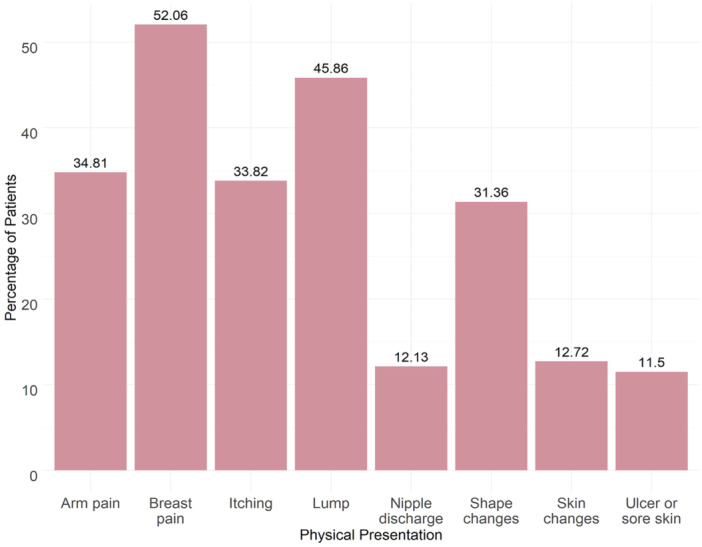
Physical presentations of the patients.

Figure 2 illustrates that the most common symptoms experienced by patients included breast discomfort (52.1%), followed by a lump (45.9%), arm discomfort (34.8%), itching (33.8%), changes in breast shape (31.4%), skin changes (12.7%), nipple discharge (12.1%), and ulcer or sore skin (11.5%).

### 3.3 Reasons for Delay in Medical Care

Table 4 identifies the primary reasons for delays in seeking medical care. The most common belief was that the issue would resolve on its own (79.1%), followed by negligence (75.5%), financial constraints (65.5%), family responsibilities (54.7%), embarrassment (44.6%), busy schedules (41.0%), and fear of a cancer diagnosis (38.1%). Additional factors included transportation difficulties (27.3%), lack of information (24.5%), delays in securing appointments (21.6%), and other reasons (20.1%).

**Table 4:** Factors contributing to patient and provider delay based on family history.

	Family history		
	Total	Yes	No
Characteristics	*n* (%)	*n* (%)	*n* (%)
Thought the problem would disappear by itself	110 (79.14)	9 (8.49)	97 (91.51)
Negligence or carelessness	105 (75.54)	8 (7.92)	93 (92.08)
Financial constraints	91 (65.47)	8 (8.99)	81 (91.01)
Competing life priorities (taking care of family)	76 (54.68)	7 (9.59)	66 (90.41)
Embarrassment about having a breast examination	62 (44.60)	5 (8.06)	57 (91.94)
Too busy (other reason)	57 (41.01)	6 (11.11)	48 (88.89)
Fear of cancer diagnosis and/or treatment	53 (38.13)	5 (9.43)	48 (90.57)
Difficult to arrange transport	38 (27.34)	4 (10.53)	34 (89.47)
Lack of information	34 (24.46)	4 (11.76)	30 (88.24)
Appointment delay	30 (21.58)	4 (13.33)	26 (86.67)
Other reason	28 (20.14)	3 (10.71)	25 (89.29)

### 3.4 Types of Delay and Associated Factors

Supporting Information S1: Table S2 highlights key risk factors for patient delay. This delay was particularly prevalent among illiterate individuals (47.6%), those with a monthly income below 5000 BDT (50.4%), and those lacking access to electronic devices (60.0%) or mass media (43.9%). Additionally, the absence of breast pain was linked to a higher rate of delay (44.1%). Adjusted analyses revealed that illiterate patients had higher odds of experiencing delay (AOR: 1.43, 95% CI: 1.17–3.05) compared to those with secondary or higher education. Patients with a monthly income of below 5,000 BDT had 3.41 (95% CI: 1.65–7.30) times higher odds of delay compared to those earning over 20,000 BDT. Conversely, those experiencing breast pain had 50% lower odds of delay (AOR: 0.50, 95% CI: 0.26–0.96) than those without pain.

Supporting Information S1: Table S3 presents significant factors associated with provider delay. A higher proportion of delay was observed among patients from rural areas (29.5%) compared to urban areas (9.8%). Geographic differences were also notable, with the highest prevalence seen in Rangpur (64.3%). Patients from the Rangpur division had over four times higher odds of experiencing provider delay (AOR: 4.60, 95% CI: 1.11–7.52) compared to those from Barisal. Rural residents had three times higher odds (AOR: 3.07, 95% CI: 1.49–6.98), and those with nipple discharge had nearly three times higher odds (AOR: 2.92, 95% CI: 1.04–8.06).

Table 5 summarizes the factors associated with total delay, higher prevalence of delay was observed among patients from Rangpur (92.9%), who were nearly six times more likely to experience delay (AOR: 5.87, 95% CI: 2.11–6.59). Delay was also more common among rural residents (60.5%), who had significantly higher odds compared to urban patients (AOR: 2.74, 95% CI: 1.40–5.52). Educational status showed a significant association, as illiterate patients had a higher prevalence of delay (61.0%) and more than twice the odds of experiencing total delay (AOR: 2.33, 95% CI: 1.09–5.11). Regarding income, patients with a monthly household income below 5,000 BDT had a higher prevalence (64.6%) and significantly increased odds of delay (AOR: 2.47, 95% CI: 1.22–5.10). Access to portable electronic devices was associated with delay, with users showing higher prevalence (54.8%) and increased odds (AOR: 1.18, 95% CI: 1.14–4.01).

**Table 5:** Prevalence and associated risk factors of total delay (*N* = 341).

	Total delay					
Characteristics	*χ* ^2^ test		Unadjusted model		Adjusted model	
	*n* (%)	*p* value	COR (95% CI)	*p* value	AOR (95% CI)	*p* value
Socioeconomic characteristics						
Age at presentation (years)						
< 40	62 (55.36)	0.960	0.82 (0.36–1.86)	0.649		
40–49	65 (54.62)		0.80 (0.35–1.80)	0.597		
50–59	44 (56.41)		0.86 (0.35–2.02)	0.736		
≥ 60	18 (60.00)		Reference			
Geographic location						
Chittagong	33 (63.46)	0.024	2.03 (1.78–5.35)	0.014	3.12 (0.94–10.85)	0.066
Dhaka	78 (52.35)		1.28 (0.56–3.00)	0.560	1.78 (0.61–5.44)	0.300
Khulna	25 (65.79)		2.24 (1.81–6.36)	0.012	3.27 (0.88–12.83)	0.080
Mymensingh	13 (39.39)		0.76 (0.27–2.15)	0.602	0.92 (0.25–3.43)	0.902
Rajshahi	10 (50.00)		1.67 (0.36–3.79)	0.796	1.89 (0.42–8.73)	0.404
Rangpur	13 (92.86)		5.17 (2.46–6.41)	0.014	5.87 (2.11–6.59)	0.020
Sylhet	3 (60.00)		1.75 (0.25–5.03)	0.573	2.81 (0.24–3.49)	0.393
Barisal	12 (46.15)		Reference		Reference	
Area of residence						
Rural	150 (60.48)	< 0.001	2.08 (1.28–3.40)	0.003	2.74 (1.40–5.52)	0.004
Urban	39 (42.39)		Reference		Reference	
Current marital status						
Single	37 (64.91)	0.115	1.60 (0.90–2.95)	0.117	1.88 (0.32–5.21)	0.503
Married	151 (53.55)		Reference		Reference	
Patient education level						
Illiterate	89 (60.96)	0.012	1.77 (1.02–3.09)	0.043	2.33 (1.09–5.11)	0.031
Primary	62 (54.39)		1.35 (0.76–2.41)	0.303	1.90 (0.90–4.04)	0.094
Secondary/Higher	37 (46.84)		Reference		Reference	
Spouse education level						
Illiterate	52 (58.43)	0.683	1.28 (0.73–2.26)	0.383		
Primary	64 (55.17)		1.12 (0.67–1.90)	0.659		
Secondary/higher	58 (52.25)		Reference			
Household monthly income (BDT)						
< 5000	73 (64.60)	0.101	1.98 (1.98–3.46)	0.015	2.47 (1.22–5.10)	0.013
5000–10,000	47 (47.96)		1.19 (0.58–2.43)	0.635	1.39 (0.58–3.38)	0.460
10,001–20,000	37 (55.22)		1.34 (0.71–2.51)	0.360	2.79 (1.23–6.57)	0.016
> 20,000	23 (52.27)		Reference		Reference	
Portable electronic devices						
Yes	173 (54.75)	0.019	1.56 (1.23–2.32)	0.020	1.18 (1.14–4.01)	0.027
No	17 (68.00)		Reference		Reference	
Mass media access						
Yes	79 (55.63)	0.979	0.99 (0.64–1.54)	0.979		
No	111 (55.78)		Reference			
Medical history of the patients						
Lump						
Yes	174 (55.59)	0.874	0.94 (0.42–2.04)	0.874		
No	16 (57.14)		Reference			
Breast pain						
Yes	46 (50.00)	0.019	1.73 (1.45–2.18)	0.019	2.34 (1.22–4.60)	0.012
No	144 (47.83)		Reference		Reference	
Nipple discharge						
Yes	14 (70.00)	0.018	1.92 (1.75–5.54)	0.018	1.72 (1.54–6.13)	0.037
No	176 (54.83)		Reference		Reference	
Skin changes						
Yes	9 (60.00)	0.733	1.20 (0.42–3.66)	0.733		
No	181 (55.52)		Reference			
Bone pain						
Yes	5 (41.67)	0.318	0.56 (0.16–1.77)	0.325		
No	185 (36.23)		Reference			
Breast self‐examination						
Yes	20 (54.05)	0.859	0.94 (0.47–1.88)	0.859		
No	164 (55.59)		Reference			
Family history of breast cancer						
Yes	15 (46.88)	0.306	0.68 (0.33–1.42)	0.308		
No	169 (56.33)		Reference			
Use of alternative or home‐based treatment						
Yes	67 (61.47)	0.098	1.48 (0.93–2.38)	0.099	1.58 (0.86–2.94)	0.141
No	114 (51.82)		Reference		Reference	
Discomfort discussing breast symptoms with spouse						
Yes	34 (62.96)	0.144	1.57 (0.86–2.93)	0.146	2.16 (1.03–4.68)	0.045
No	120 (51.95)		Reference		Reference	
History of prior clinical breast examination						
Yes	8 (50.00)	0.647	0.79 (0.28–2.20)	0.647		
No	177 (44.16)		Reference			
Type of first healthcare facility visited						
Others	9 (69.23)	0.599	1.87 (0.58–7.18)	0.118	1.01 (0.56–1.82)	0.966
Private hospital	71 (55.73)		1.04 (0.67–1.64)	0.844	0.97 (0.21–1.37)	0.967
Government hospital	107 (54.62)		Reference		Reference	

Abbreviations: AOR, adjusted odds ratio; CI, confidence interval; COR, crude odds ratio.

In terms of clinical characteristics, patients with breast pain had significantly higher odds of delay (AOR: 2.34, 95% CI: 1.22–4.60), and those with nipple discharge showed a higher prevalence (70.0%) along with increased odds (AOR: 1.72, 95% CI: 1.54–6.13). Patients who felt discomfort discussing breast symptoms with their spouse had a higher prevalence of delay (63.0%) and were more than twice as likely to experience total delay (AOR: 2.16, 95% CI: 1.03–4.68).

## 4 Discussion

This study highlights several key factors contributing to diagnostic delays in breast cancer care in Bangladesh, including self‐perceived beliefs and negligence, fear, embarrassment, economic and educational barriers, and geographic disparities. Understanding these factors is critical for developing effective interventions to reduce delays and improve outcomes for breast cancer patients.

A significant portion of patients (79.1%) delayed seeking medical attention due to the belief that their symptoms would resolve on their own. This suggests a pervasive lack of awareness about the severity of breast cancer symptoms, such as lumps or nipple discharge, which often leads to advanced‐stage diagnoses [30]. This self‐perceived belief is dangerous as it allows the disease to progress unchecked, increasing the risk of mortality. Negligence or carelessness was also cited by 75.5% of participants, indicating that even when symptoms are acknowledged, there is a tendency to delay seeking care due to underestimating the potential severity of the symptoms. This finding aligns with similar studies conducted in Tunisia and Libya, where a significant percentage of women delayed seeking medical help due to the perception that their symptoms were not serious [8, 31]. Improving awareness and education about the early warning signs of breast cancer is essential to encourage prompt medical consultation and reduce patient delay.

Fear of a cancer diagnosis or treatment emerged as a critical factor in delaying medical consultation, reported by 38.1% of participants. This fear is often compounded by a lack of knowledge and misconceptions about the disease and its treatment. Negative perceptions about the toxicity of cancer treatments, such as chemotherapy, can deter patients from seeking help. This is particularly evident in developing countries where fear of diagnosis is more prevalent among those with a family history of breast cancer [8, 32]. In contrast, in the UK, only 4.9% of delays are attributed to fear, highlighting a significant difference in perception between developed and developing nations [33]. Disseminating positive information about cancer survivorship and organizing awareness campaigns can help alleviate this fear. Furthermore, encouraging cancer survivors to share their experiences could normalize discussions about the disease and reduce the stigma associated with cancer diagnosis and treatment.

Embarrassment related to breast examinations and discussing reproductive health is another significant barrier, particularly in societies with conservative norms. In Bangladesh, where concepts like “Purdah” (seclusion/veiling) promote modesty, women may feel uncomfortable undergoing breast examinations or discussing their symptoms with healthcare providers. This cultural barrier is evident in our study, where 44.6% of participants reported embarrassment as a reason for delay. Similar trends are observed among South Asian women residing in developed countries, indicating that cultural influences on health‐seeking behavior persist even outside their native contexts [34, 35]. Efforts to increase awareness and normalize breast health discussions are crucial. Training healthcare providers, particularly female staff, to handle these situations sensitively can also reduce patient discomfort and encourage timely medical consultations. In conservative societies such as Bangladesh, sociocultural norms, including Purdah, restrict women's autonomy and mobility, limiting timely healthcare‐seeking [36, 37]. Gender dynamics, stigma surrounding breast‐related symptoms, and fear of social consequences further exacerbate delays [38].

Socio‐economic factors, including education level and household income, significantly impact patient delays. Our study found that patients with a monthly income of 5000–10,000 BDT had 1.45 times higher odds of experiencing delays compared to those with incomes over 20,000 BDT. Financial constraints often prevent patients from seeking timely medical care, as many may prioritize other family needs over personal health. Moreover, education plays a crucial role in health‐seeking behavior. Illiterate patients were found to delay diagnosis at twice the rate of those with secondary education or higher. Educated women are more likely to recognize symptoms and seek medical attention promptly, which is consistent with findings from other studies [39, 40]. Lack of access to electronic devices and mass media further exacerbates this issue, as these are primary sources of health information. Women without access to these resources are less likely to be aware of breast cancer symptoms and the importance of early detection [41, 42]. Targeted interventions, such as community‐based educational programs and mass media campaigns, are needed to address these barriers.

Geographic location and area of residence significantly contribute to provider delays. Patients from rural areas and the Rangpur division experienced longer diagnosis times due to limited access to healthcare resources, long waiting times, and a scarcity of specialized medical professionals. In Bangladesh, there are approximately two nurses and five physicians per 10,000 people, which severely limits the capacity to provide timely care, particularly in rural areas [15]. In Bangladesh, cancer services are highly centralized, which creates bottlenecks and delays for rural populations [43]. Our findings are consistent with Muyisa et al., who reported similar delays driven by financial barriers, limited awareness, and health system inefficiencies in African settings [44]. Their study further quantified delay intervals and highlighted referral bottlenecks and diagnostic capacity gaps, which align with our observations. Beyond geographic disparities, systemic inefficiencies play a critical role in provider delays. These include limited diagnostic infrastructure, inadequate referral coordination between primary and tertiary care, and prolonged waiting times at specialized facilities [44]. A systematic review identified deficiencies in primary healthcare and referral processes as major factors influencing provider delays in breast cancer diagnosis [10]. Moreover, most specialized healthcare facilities are located in urban areas, forcing rural patients to rely on primary healthcare centers that may not be equipped for early cancer detection [41]. Training healthcare providers in early detection and improving referral systems are essential for reducing provider delays [45].

### 4.1 Strengths and Limitations of the Study

This study has a number of noteworthy advantages. It gathers information from a population representative of Bangladesh's socioeconomically disadvantaged groups and is carried out at the National Institute of Cancer Research and Hospital (NICRH). The study provides a thorough understanding of the obstacles to prompt breast cancer care by looking at a variety of sociodemographic, clinical, emotional, and system‐level factors. Cultural sensitivity was ensured by using trained female interviewers, enhancing response accuracy. The reliability and contextual relevance of the data gathered are further reinforced by the use of a validated, pilot‐tested questionnaire. There are several limitations to this study. First, the study was conducted in two public hospitals in Dhaka, without including private healthcare facilities. This limits the generalizability of the findings to the broader population, as private hospitals may have different patient demographics and healthcare delivery models. Second, the lack of a systematic referral system and organized cancer screening programs in Bangladesh hampers early detection efforts, and cancer staging data were not comprehensively available for all patients, limiting the analysis of the impact of delays on cancer progression. Additionally, the use of a non‐random convenience sampling method may introduce selection bias, and recall bias may affect the accuracy of self‐reported data on symptom onset and first medical consultation. Recall bias may have affected the accuracy of reported timelines, although efforts were made to validate responses using medical records. In this study, detailed health system–level factors (e.g., referral systems, provider availability, and diagnostic capacity) were not captured and should be explored in future research. However, we included several health‐seeking behavioral and sociocultural variables (alternative treatment or prior clinical breast examination) to provide insight into the broader context of delays.

## 5 Conclusions

According to this study, 41% of Bangladeshi women experience patient delays, 24.3% experience provider delays, and 55.7% experience total delays longer than 4 months in the diagnosis and treatment of breast cancer. Low income and educational attainment, living in a rural area, having limited access to healthcare, and emotional and cultural barriers like fear, carelessness, and embarrassment were all strongly linked to these delays. The study emphasizes how urgently focused interventions are needed to lessen these delays. Campaigns to raise awareness of early breast cancer symptoms and the value of timely medical consultation are crucial, particularly in underserved and rural areas. Improving early diagnosis and treatment outcomes requires growing organized screening programs and educating healthcare professionals about early detection. Priorities should also be given to removing socioeconomic barriers, enhancing healthcare accessibility, and putting in place a strong referral system to expedite patient management. Collaborations with community health workers and non‐governmental organizations can be extremely important for patient support and outreach. Reducing delays in breast cancer care in Bangladesh requires a comprehensive, multi‐level approach. This includes improving education for both patients and healthcare providers to promote early symptom recognition, expanding access to affordable and timely care, and implementing community‐based awareness programs led by female health workers. Additionally, decentralizing screening services through mobile clinics can help overcome geographic barriers, while strengthening primary care referral systems and adopting task‐shifting strategies can facilitate earlier diagnosis. Financial protection mechanisms are also essential to reduce economic barriers and improve access to treatment.

## Author Contributions

**Mohammad Sorowar Hossain:** conceptualization; investigation, writing – original draft, writing – review and editing, resources, and supervision. **Mohammad Nayeem Hasan:** validation, visualization, writing – review and editing, formal analysis, writing – original draft, data curation, and methodology. **Sumaya Khan Trisha:** writing – original draft, writing – review and editing. **Md. Waheed Akhter:** investigation, conceptualization, supervision, writing – review and editing.

## Funding

The authors have nothing to report.

## Ethics Statement

This study protocol was approved by the Ethical Review Board of the National Institute of Cancer Research and Health (NICRH/Ethics/2017/29). Informed written consent was taken from each patient. Trained research assistants used a structured questionnaire to conduct in‐person interviews with illiterate respondents in order to gather data. The interviewer recorded the responses after reading the questions out loud in the local tongue. A literate witness, typically a family member or caregiver who was with the patient, signed the consent form on the participant's behalf after verbally obtaining informed consent. The ethics committee approved this procedure, which made sure that participants understood the goals and methods of the study before they participated.

## Conflicts of Interest

The authors declare no conflicts of interest.

## Transparency Statement

The corresponding author, Mohammad Sorowar Hossain, affirms that this manuscript is an honest, accurate, and transparent account of the study being reported; that no important aspects of the study have been omitted; and that any discrepancies from the study as planned (and, if relevant, registered) have been explained.

## Supporting information

Supporting File

## Data Availability

The data sets used and/or analyzed during the current study are available from the corresponding author on reasonable request.
