# Supplementary material for: Understanding Delays in Breast Cancer Diagnosis in Bangladesh: A Facility‐Based Cross‐Sectional Study
Source: Health Sci Rep. 2026 Jun 28;9(7):e72718. doi: 10.1002/hsr2.72718 (PMC13311312; doi:10.1002/hsr2.72718)
Supplement: Supplementary file 1 — Supporting File [file HSR2-9-e72718-s001.docx]

**Supplementary Information**

**Table S1** STROBE Statement—Checklist of items that should be included in reports of *cross-sectional studies.*

|  | **Item No** | **Recommendation** | **Page # in manuscript** |
| --- | --- | --- | --- |
| **Title and abstract** | 1 | (*a*) Indicate the study’s design with a commonly used term in the title or the abstract | 2 |
|  |  | (*b*) Provide in the abstract an informative and balanced summary of what was done and what was found | 2 |
| **Introduction** | | |  |
| Background/rationale | 2 | Explain the scientific background and rationale for the investigation being reported | 3 |
| Objectives | 3 | State specific objectives, including any prespecified hypotheses | 4 |
| **Methods** | | |  |
| Study design | 4 | Present key elements of study design early in the paper | 4 |
| Setting | 5 | Describe the setting, locations, and relevant dates, including periods of recruitment, exposure, follow-up, and data collection | 5 |
| Participants | 6 | (*a*) Give the eligibility criteria, and the sources and methods of selection of participants | 5 |
| Variables | 7 | Clearly define all outcomes, exposures, predictors, potential confounders, and effect modifiers. Give diagnostic criteria, if applicable | 6-7 |
| Data sources/ measurement | 8* | For each variable of interest, give sources of data and details of methods of assessment (measurement). Describe comparability of assessment methods if there is more than one group | 6-7 |
| Bias | 9 | Describe any efforts to address potential sources of bias | N/A |
| Study size | 10 | Explain how the study size was arrived at | N/A |
| Quantitative variables | 11 | Explain how quantitative variables were handled in the analyses. If applicable, describe which groupings were chosen and why | 7 |
| Statistical methods | 12 | (*a*) Describe all statistical methods, including those used to control for confounding | 7 |
|  |  | (*b*) Describe any methods used to examine subgroups and interactions | N/A |
|  |  | (*c*) Explain how missing data were addressed | N/A |
|  |  | (*d*) If applicable, describe analytical methods taking account of sampling strategy | N/A |
|  |  | (*e*) Describe any sensitivity analyses | 8-9 |
| **Results** | | |  |
| Participants | 13* | (a) Report numbers of individuals at each stage of study—eg numbers potentially eligible, examined for eligibility, confirmed eligible, included in the study, completing follow-up, and analysed | N/A |
|  |  | (b) Give reasons for non-participation at each stage | N/A |
|  |  | (c) Consider use of a flow diagram |  |
| Descriptive data | 14* | (a) Give characteristics of study participants (eg demographic, clinical, social) and information on exposures and potential confounders | 7-8 |
|  |  | (b) Indicate number of participants with missing data for each variable of interest | N/A |
| Outcome data | 15* | Report numbers of outcome events or summary measures | 7-8 |
| Main results | 16 | (*a*) Give unadjusted estimates and, if applicable, confounder-adjusted estimates and their precision (eg, 95% confidence interval). Make clear which confounders were adjusted for and why they were included | 8-9 |
|  |  | (*b*) Report category boundaries when continuous variables were categorized | N/A |
|  |  | (*c*) If relevant, consider translating estimates of relative risk into absolute risk for a meaningful time period | N/A |
| Other analyses | 17 | Report other analyses done—eg analyses of subgroups and interactions, and sensitivity analyses | 8-9 |
| **Discussion** | | |  |
| Key results | 18 | Summarise key results with reference to study objectives | 9-11 |
| Limitations | 19 | Discuss limitations of the study, taking into account sources of potential bias or imprecision. Discuss both direction and magnitude of any potential bias | 11 |
| Interpretation | 20 | Give a cautious overall interpretation of results considering objectives, limitations, multiplicity of analyses, results from similar studies, and other relevant evidence | 12 |
| Generalisability | 21 | Discuss the generalisability (external validity) of the study results | N/A |
| **Other information** | | |  |
| Funding | 22 | Give the source of funding and the role of the funders for the present study and, if applicable, for the original study on which the present article is based | Title page |

*Give information separately for exposed and unexposed groups.

**Note:** An Explanation and Elaboration article discusses each checklist item and gives methodological background and published examples of transparent reporting. The STROBE checklist is best used in conjunction with this article (freely available on the Web sites of PLoS Medicine at http://www.plosmedicine.org/, Annals of Internal Medicine at http://www.annals.org/, and Epidemiology at http://www.epidem.com/). Information on the STROBE Initiative is available at www.strobe-statement.org.

**Table S2: Prevalence and associated risk factors of patient delay (N=339)**

|  | **Patient delay** | | | | | |
| --- | --- | --- | --- | --- | --- | --- |
| **Characteristics** | **Chi-square test** | | **Unadjusted model** | | **Adjusted model** | |
|  | **n (%)** | ***P-value*** | **COR (95% CI)** | ***P-value*** | **AOR (95% CI)** | ***P-value*** |
| **Socioeconomic characteristics** |  |  |  |  |  |  |
| ***Age at presentation (years)*** |  |  |  |  |  |  |
| <40 | 44 (39.29) | 0.839 | 1.12 (0.49 – 2.64) | 0.794 |  |  |
| 40-49 | 52 (44.07) |  | 1.36 (0.60 – 3.19) | 0.465 |  |  |
| 50-59 | 31 (40.26) |  | 1.16 (0.49 – 2.85) | 0.733 |  |  |
| ≥60 | 11 (36.67) |  | Reference |  |  |  |
| ***Geographic location*** |  |  |  |  |  |  |
| Chittagong | 22 (43.14) | 0.669 | 1.21 (0.47 – 3.25) | 0.694 |  |  |
| Dhaka | 61 (40.94) |  | 1.11 (0.48 – 2.68) | 0.812 |  |  |
| Khulna | 19 (50.00) |  | 1.60 (0.59 – 4.51) | 0.364 |  |  |
| Mymensingh | 9 (27.27) |  | 0.60 (0.20 – 1.80) | 0.363 |  |  |
| Rajshahi | 8 (40.00) |  | 1.07 (0.32 – 3.54) | 0.916 |  |  |
| Rangpur | 5 (35.71) |  | 0.89 (0.22 – 3.39) | 0.864 |  |  |
| Sylhet | 3 (60.00) |  | 2.40 (0.34 – 10.77) | 0.380 |  |  |
| Barisal | 10 (38.46) |  | Reference |  |  |  |
| ***Area of residence*** |  |  |  |  |  |  |
| Rural | 106 (42.91) | 0.270 | 1.32 (0.81 – 2.19) | 0.271 |  |  |
| Urban | 33 (36.26) |  | Reference |  |  |  |
| ***Current marital status*** |  |  |  |  |  |  |
| Single | 111 (48.21) | 0.226 | 1.42 (0.80 – 2.54) | 0.226 |  |  |
| Married | 27 (39.50) |  | Reference |  |  |  |
| ***Patient education level*** |  |  |  |  |  |  |
| Illiterate | 69 (47.59) | 0.042 | 1.82 (1.03 – 3.25) | 0.021 | 1.43 (1.17 - 3.05) | 0.035 |
| Primary | 43 (37.72) |  | 1.21 (0.66 – 2.23) | 0.534 | 1.44 (0.68 - 3.08) | 0.348 |
| Secondary/Higher | 26 (33.33) |  | Reference |  | Reference |  |
| ***Spouse education level*** |  |  |  |  |  |  |
| Illiterate | 39 (44.32) | 0.637 | 1.29 (0.73 – 2.28) | 0.383 |  |  |
| Primary | 45 (38.79) |  | 1.03 (0.60 – 1.76) | 0.925 |  |  |
| Secondary/Higher | 42 (38.18) |  | Reference |  |  |  |
| ***Household monthly income (BDT)*** |  |  |  |  |  |  |
| <5000 | 57 (50.44) | 0.011 | 2.64 (1.49 – 4.75) | <0.001 | 3.41 (1.65 - 7.30) | <0.001 |
| 5000-10000 | 27 (27.84) |  | 1.97 (0.93 – 4.16) | 0.074 | 2.15 (0.84 - 5.46) | 0.106 |
| 10001-20000 | 28 (41.79) |  | 1.86 (0.97 – 3.61) | 0.064 | 2.73 (1.23 - 6.21) | 0.014 |
| >20000 | 19 (43.18) |  | Reference |  | Reference |  |
| ***Portable electronic devices*** |  |  |  |  |  |  |
| Yes | 124 (39.49) | 0.045 | 0.44 (0.18 – 0.99) | 0.049 | 0.79 (0.54 - 1.71) | 0.094 |
| No | 15 (60.00) |  | Reference |  | Reference |  |
| ***Mass media access*** |  |  |  |  |  |  |
| Yes | 52 (36.88) | 0.193 | 0.75 (0.48 – 1.16) | 0.193 | 0.86 (0.45 - 1.64) | 0.647 |
| No | 87 (43.94) |  | Reference |  | Reference |  |
| **Medical history of the patients** |  |  |  |  |  |  |
| ***Lump*** |  |  |  |  |  |  |
| Yes | 128 (41.16) | 0.847 | 1.08 (0.50 – 2.45) | 0.847 |  |  |
| No | 11 (39.39) |  | Reference |  |  |  |
| ***Breast pain*** |  |  |  |  |  |  |
| Yes | 30 (32.61) | 0.045 | 0.61 (0.37 – 1.01) | 0.055 | 0.50 (0.26 - 0.96) | 0.041 |
| No | 109 (44.13) |  | Reference |  | Reference |  |
| ***Nipple discharge*** |  |  |  |  |  |  |
| Yes | 8 (42.11) | 0.920 | 1.05 (0.40 – 2.66) | 0.920 |  |  |
| No | 131 (40.94) |  | Reference |  |  |  |
| ***Skin changes*** |  |  |  |  |  |  |
| Yes | 7 (46.67) | 0.648 | 1.27 (0.44 – 3.63) | 0.649 |  |  |
| No | 132 (40.74) |  | Reference |  |  |  |
| ***Bone pain*** |  |  |  |  |  |  |
| Yes | 4 (33.33) | 0.582 | 0.71 (0.19 – 2.31) | 0.584 |  |  |
| No | 135 (41.28) |  | Reference |  |  |  |
| ***Breast self-examination*** |  |  |  |  |  |  |
| Yes | 15 (40.54) | 0.975 | 1.01 (0.50 – 2.02) | 0.975 |  |  |
| No | 118 (40.27) |  | Reference |  |  |  |
| ***Family history of breast cancer*** |  |  |  |  |  |  |
| Yes | 11 (34.38) | 0.450 | 0.75 (0.34 – 1.57) | 0.451 |  |  |
| No | 123 (41.28) |  | Reference |  |  |  |
| ***Use of alternative or home-based treatment*** |  |  |  |  |  |  |
| Yes | 56 (51.38) | 0.005 | 1.93 (1.21-3.09) | 0.005 | 2.77 (1.53 - 5.11) | <0.001 |
| No | 77 (35.32) |  | Reference |  |  |  |
| ***Discomfort discussing breast symptoms with spouse*** |  |  |  |  |  |  |
| Yes | 26 (50.0) | 0.078 | 1.72 (0.94-3.16) | 0.080 | 1.34 (0.63 - 2.81) | 0.142 |
| No | 85 (36.8) |  | Reference |  |  |  |
| ***History of prior clinical breast examination*** |  |  |  |  |  |  |
| Yes | 3 (18.75) | 0.069 | 0.32 (0.27-1.03) | 0.083 | 0.40 (0.28 - 1.40) | 0.186 |
| No | 131 (41.59) |  | Reference |  |  |  |
| ***Type of first healthcare facility visited*** |  |  |  |  |  |  |
| Others | 8 (61.54) | 0.324 | 2.22 (0.70-7.70) | 0.181 | 2.26 (0.51 - 8.10) | 0.297 |
| Private hospital | 77 (40.31) |  | 0.94 (0.60-1.48) | 0.783 | 0.84 (0.47 - 1.50) | 0.549 |
| Government hospital | 54 (41.86) |  | Reference |  |  |  |

AOR: adjusted odds ratio, CI: confidence interval, COR: crude odds ratio

**Table S3: Prevalence and associated risk factors of provider delay (N=337)**

|  | **Provider delay** | | | | | |
| --- | --- | --- | --- | --- | --- | --- |
| **Characteristics** | **Chi-square test** | | **Unadjusted model** | | **Adjusted model** | |
|  | **n (%)** | ***P-value*** | **COR (95% CI)** | ***P-value*** | **AOR (95% CI)** | ***P-value*** |
| **Socioeconomic characteristics** |  |  |  |  |  |  |
| ***Age at presentation (years)*** |  |  |  |  |  |  |
| <40 | 23 (20.54) | 0.613 | 0.57 (0.23 – 1.48) | 0.233 |  |  |
| 40-49 | 29 (24.37) |  | 0.72 (0.30 – 1.81) | 0.463 |  |  |
| 50-59 | 20 (26.67) |  | 0.81 (0.32 – 2.13) | 0.656 |  |  |
| ≥60 | 9 (1.03) |  | Reference |  |  |  |
| ***Geographic location*** |  |  |  |  |  |  |
| Chittagong | 14 (26.08) | 0.030 | 1.23 (0.42 – 3.91) | 0.714 | 1.17 (0.38 – 3.85) | 0.789 |
| Dhaka | 27 (18.49) |  | 0.76 (0.29 – 2.23) | 0.585 | 0.82 (0.30 – 2.49) | 0.704 |
| Khulna | 10 (26.32) |  | 1.19 (0.38 – 4.00) | 0.769 | 1.13 (0.35 – 3.94) | 0.838 |
| Mymensingh | 8 (25.00) |  | 1.11 (0.33 – 3.88) | 0.865 | 1.14 (0.33 – 4.14) | 0.837 |
| Rajshahi | 6 (30.00) |  | 1.43 (0.37 – 5.49) | 0.597 | 1.48 (0.37 – 5.92) | 0.575 |
| Rangpur | 9 (64.29) |  | 6.00 (1.51 – 7.06) | 0.014 | 4.60 (1.11 – 7.52) | 0.041 |
| Sylhet | 1 (20.00) |  | 0.83 (0.04 – 7.14) | 0.880 | 0.85 (0.04 – 7.58) | 0.894 |
| Barisal | 6 (23.08) |  | Reference |  | Reference |  |
| ***Area of residence*** |  |  |  |  |  |  |
| Rural | 72 (29.51) | <0.001 | 3.86 (1.93 – 8.62) | <0.001 | 3.07 (1.49 – 6.98) | 0.004 |
| Urban | 9 (9.78) |  | Reference |  | Reference |  |
| ***Current marital status*** |  |  |  |  |  |  |
| Single | 17 (30.36) | 0.213 | 1.49 (0.78 – 2.79) | 0.215 |  |  |
| Married | 63 (22.58) |  | Reference |  |  |  |
| ***Patient education level*** |  |  |  |  |  |  |
| Illiterate | 36 (24.66) | 0.916 | 1.14 (0.59 – 2.23) | 0.704 |  |  |
| Primary | 28 (24.78) |  | 1.14 (0.58 – 2.31) | 0.703 |  |  |
| Secondary/Higher | 17 (22.37) |  | Reference |  |  |  |
| ***Spouse education level*** |  |  |  |  |  |  |
| Illiterate | 18 (20.22) | 0.283 | 0.94 (0.46 – 1.87) | 0.854 |  |  |
| Primary | 33 (28.70) |  | 1.49 (0.81 – 2.77) | 0.204 |  |  |
| Secondary/Higher | 23 (21.30) |  | Reference |  |  |  |
| ***Household monthly income (BDT)*** |  |  |  |  |  |  |
| <5000 | 28 (24.78) | 0.791 | 1.36 (0.58 – 3.47) | 0.500 |  |  |
| 5000-10000 | 27 (27.55) |  | 1.57 (0.67 – 4.03) | 0.322 |  |  |
| 10001-20000 | 16 (23.88) |  | 1.29 (0.51 – 3.51) | 0.597 |  |  |
| >20000 | 8 (19.51) |  | Reference |  |  |  |
| ***Portable electronic devices*** |  |  |  |  |  |  |
| Yes | 78 (24.76) | 0.487 | 1.48 (0.53 – 5.24) | 0.489 |  |  |
| No | 4 (18.18) |  | Reference |  |  |  |
| ***Mass media access*** |  |  |  |  |  |  |
| Yes | 38 (26.76) | 0.375 | 1.25 (0.76 – 2.07) | 0.376 |  |  |
| No | 44 (22.56) |  | Reference |  |  |  |
| **Medical history of the patients** |  |  |  |  |  |  |
| ***Lump*** |  |  |  |  |  |  |
| Yes | 75 (24.04) | 0.657 | 0.81 (0.34 – 2.16) | 0.657 |  |  |
| No | 7 (28.00) |  | Reference |  |  |  |
| ***Breast pain*** |  |  |  |  |  |  |
| Yes | 28 (30.43) | 0.110 | 1.55 (0.90 – 2.64) | 0.111 | 1.36 (0.76 – 2.41) | 0.296 |
| No | 54 (22.04) |  | Reference |  | Reference |  |
| ***Nipple discharge*** |  |  |  |  |  |  |
| Yes | 9 (45.00) | 0.026 | 2.73 (1.06 – 6.86) | 0.032 | 2.92 (1.04 – 8.06) | 0.037 |
| No | 73 (23.03) |  | Reference |  | Reference |  |
| ***Skin changes*** |  |  |  |  |  |  |
| Yes | 2 (13.33) | 0.310 | 0.46 (0.07 – 1.73) | 0.321 |  |  |
| No | 80 (24.84) |  | Reference |  |  |  |
| ***Bone pain*** |  |  |  |  |  |  |
| Yes | 1 (8.33) | 0.188 | 0.27 (0.01 – 1.44) | 0.218 |  |  |
| No | 81 (24.92) |  | Reference |  |  |  |
| ***Breast self-examination*** |  |  |  |  |  |  |
| Yes | 8 (21.62) | 0.638 | 0.82 (0.38 – 1.80) | 0.638 |  |  |
| No | 74 (25.17) |  | Reference |  |  |  |
| ***Family history of breast cancer*** |  |  |  |  |  |  |
| Yes | 7 (21.88) | 0.750 | 0.87 (0.33 – 2.00) | 0.750 |  |  |
| No | 73 (24.41) |  | Reference |  |  |  |
| ***Use of alternative or home-based treatment*** |  |  |  |  |  |  |
| Yes | 24 (22.02) | 0.537 | 0.84 (0.48-1.44) | 0.537 |  |  |
| No | 55 (25.11) |  | Reference |  |  |  |
| ***Discomfort discussing breast symptoms with spouse*** |  |  |  |  |  |  |
| Yes | 12 (23.53) | 0.914 | 0.96 (0.45-1.92) | 0.914 |  |  |
| No | 56 (24.24) |  | Reference |  |  |  |
| ***History of prior clinical breast examination*** |  |  |  |  |  |  |
| Yes | 5 (31.25) | 0.533 | 1.41 (0.43-4.01) | 0.535 |  |  |
| No | 77 (24.37) |  | Reference |  |  |  |
| ***Type of first healthcare facility visited*** |  |  |  |  |  |  |
| Others | 3 (23.08) | 0.997 | 0.96 (0.21-3.38) | 0.953 |  |  |
| Private hospital | 46 (23.96) |  | 1.01 (0.60-1.72) | 0.976 |  |  |
| Government hospital | 30 (23.81) |  | Reference |  |  |  |

AOR: adjusted odds ratio, CI: confidence interval, COR: crude odds ratio

# Study Questionnaire

1. **Patient's Name**

- Open-ended

2. **Contact Telephone**

- Open-ended

3. **Age (in years)**

- Integer, range: 18-99

4. **Home District (Permanent Residence)**

- Dropdown list of districts

5. **Location of Residence**

- Rural / Urban

6. **Current Place of Residence (District)**

- Dropdown list of districts

7. **Education Completed**

- Illiterate / Primary / Secondary / Higher Secondary (College) / Graduate

8. **Marital Status**

- Single / Widowed / Never Married / Married

9. **Husband's Education (if applicable)**

- Illiterate / Primary / Secondary / Higher Secondary (College) / Graduate

10. **Monthly Family Income in Taka**

- <5,000 / 10,000 / 20,000 / Others

11. **Access to Communication and Media (select all that apply)**

- Social media / Mobile / Smartphone / Personal Computer / TV / Newspaper

12. **Family History of Breast Cancer**

- Yes / No

13. **When did you first realize the problem with your breast?**

- Date field

14. **What was the first symptom noticed? (select all that apply)**

- Lump / Skin changes / Breast pain / Nipple discharge / Bone pain / Other

15. **When you noticed the symptom, did you think it could be cancer?**

- Yes / No

16. **Have you experienced any of the following discomforts? (Yes/No)**

- Lump in armpit, neck, or trunk

- Pain in breast

- Pain in arm

- Color changes in breast skin

- Ulcer or sore on breast skin

- Itching in the breast

- Changes in breast shape

- Liquid or blood discharge from nipple

17. **When did you visit the doctor after realizing the problem? **

- Date field

18. **Reasons for not visiting the doctor initially: (Yes/No)**

- Thought problem would disappear

- Fear/Too scared

- Too embarrassed

- Negligence/Carelessness

- Taking care of family

- Too busy

- Lack of money

- Difficulty arranging transport

- Didn't know where to go

- Difficulty making an appointment

- Other

19. **Medical center visited before cancer treatment center**

- Private clinic/hospital / General hospital / Upazila health complex / NGO clinic / Pharmacy / Other

20. **When did you receive the final diagnosis or begin treatment after your initial medical consultation? **

- Date field

21. **Did you try any alternative remedies?**

- Yes / No

(If yes: Homeopathy / Kobiraj / Jharfuk / Other)

22. **Who did you talk to first about your health problem?**

- Husband / Mother / Sibling / Friend / Neighbor / Other

23. **Who recommended consulting with a doctor?**

- Husband / Mother / Sibling / Friend / Neighbor / Other

24. **Did you feel fear or discomfort telling your spouse?**

- Yes / No

25. **Did you receive support from your spouse after diagnosis?**

- Yes / No

(If no, did you experience negative behavior from spouse?)

26. **Did you receive support from your social circle?**

- Yes / No

27. **Do you usually check your own breasts?**

- Yes / No

28. **Did a doctor or nurse check your breasts before this health problem?**

- Yes / No

29. **Had you heard of mammography or mammogram before?**

- Yes / No

30. **Do you know someone close who had or has cancer?**

- Yes / No

31. **Did you know about breast cancer before?**

- Yes / No

32. **Is there any particular information you wish you knew and want others to know?**

- Open-ended

33. **Stage of Cancer**

- Stage I / Stage II / Stage III / Stage IV
